# Long-term oncologic outcomes after laparoscopic vs. open colon cancer resection: a high-quality population-based analysis in a Southern German district

**DOI:** 10.1007/s00464-018-6158-4

**Published:** 2018-03-30

**Authors:** Vinzenz Völkel, Teresa Draeger, Michael Gerken, Monika Klinkhammer-Schalke, Alois Fürst

**Affiliations:** 1 Universität Regensburg, Universitätsstraße 31, 93053 Regensburg, Germany; 2 Tumorzentrum Regensburg - Institut für Qualitätssicherung und Versorgungsforschung der Universität Regensburg, Am BioPark 9, 93053 Regensburg, Germany; 3 Caritas Krankenhaus St. Josef Regensburg, Klinik für Allgemein-, Viszeral-, Thoraxchirurgie und Adipositasmedizin, Landshuter Str. 65, 93053 Regensburg, Germany

**Keywords:** Bowel cancer, Minimal invasive surgery, Health services research, Registries, Cohort studies

## Abstract

**Background:**

Over 20 years after the introduction of laparoscopic surgery for colon cancer, many surgeons still prefer the open approach. Whereas randomized controlled trials (RCTs) have proven the oncologic safety of laparoscopy, long-term data depicting daily clinical routine are scarce.

**Methods:**

This population-based cohort study compares 5-year overall, relative, and recurrence-free survival rates after laparoscopic and open colon carcinoma surgery. Data derive from an independent German cancer registry encompassing all tumor patients within a political district of 1.1 million inhabitants. The final analysis included 2669 patients with major elective resection of primary non-metastatic colonic adenocarcinoma between January 1, 2004 and December 31, 2013. Survival rates were compared using Kaplan–Meier analyses, relative survival models, and multivariate Cox regression. Sensitivity analysis quantified selection bias.

**Results:**

The proportion of laparoscopic procedures increased from 9.7 to 25.8% in 2011 and dropped again to 15.8% at the end of observation period. Laparoscopy patients were younger, had a lower tumor stage, and were more likely to receive postoperative chemotherapy. Overall, relative, and recurrence-free survival was significantly superior or equivalent in Kaplan–Meier analysis (5-year overall survival rate open vs. laparoscopic: 69.0 vs. 80.2%, *p* < 0.001). The superiority of laparoscopy mostly remained stable after adjusting for confounders, although significance was only reached in T1-3 patients without lymph node metastases (overall survival: hazard ratio (HR) 0.654; 95% confidence interval (CI) 0.446–0.958; *p* = 0.029).

**Conclusion:**

Laparoscopy is a safe and promising alternative to the open approach in daily clinic practice. These favorable outcomes require future confirmation by high-quality studies outside the setting of RTCs.

## Background

Due to increasing life expectancy [1], neoplastic diseases are gaining importance worldwide. One out of ten tumors are located in the colon or rectum, thus rendering colorectal carcinomas the third most common cancer within the male and the second most common tumor within the female population [2]. During past years, different randomized controlled trials (RCTs) have proven the oncologic safety of laparoscopy [3]. However, there still exists a great deal of skepticism concerning the external validity of these results in real-life situations, and reliable population-based studies addressing long-term survival are scarce. In Germany, colorectal cancer treatment is highly standardized by national evidence-based guidelines, which ensure a patient’s optimal treatment regardless of their social or economic status [4]. This provides truly objective conditions for examining oncologic outcomes after tumor resection in daily clinical practice. As one of the biggest national cancer registries, the independent University of Regensburg Institute for Quality Control and Health Services Research [5] meticulously monitors the treatment process and outcomes of all cancer patients within a cohesive population of 1.1 million people [6], and thus guarantees representative results.

## Patients and methods

This retrospective cohort study aims to compare overall, relative, and recurrence-free survival rates after laparoscopic and open colon carcinoma surgery. Data derive from an official cancer registry (Tumor Center Regensburg/University of Regensburg Institute for Quality Control and Health Services Research), which systematically collects medical records of all tumor patients registered within a large political district in southern Germany. Information on each patient includes demographics, tumor characteristics, surgical procedure, postoperative (adjuvant) chemotherapy, and other malignant neoplasms, if applicable (Table 1). In compliance with German data protection laws, all concerned persons have to formally consent to anonymized use of their data. To obtain actual information on life status, a regular exchange with local registration offices takes place.

**Table 1:** Baseline characteristics of the study population according to surgical access

	Open (*n* = 2283)		Laparoscopic (*n* = 386)		Chi-square
	*n*	%	*n*	%	*p* Value
*Gender*					
Male	1263	55.3	221	57.3	0.480
Female	1020	44.7	165	42.7	
*Age*					
≤ 64	561	24.6	153	39.6	< 0.001
65–77	1030	45.1	164	42.5	
≥ 78	692	30.3	69	17.9	
*Previous carcinomas*					
No	2169	95.0	377	97.7	0.021
Yes	114	5.0	9	2.3	
*Synchronous carcinomas*					
No	2223	97.4	380	98.4	0.209
Yes	60	2.6	6	1.6	
*Grading*					
G1/2	1806	79.1	324	83.9	0.029
G3/4	477	20.9	62	16.1	
*UICC stage*					
I	546	23.9	152	39.4	< 0.001
II	973	42.6	120	31.1	
III	764	33.5	114	29.5	
*T-stage*					
T1-3	1933	84.7	359	93.0	< 0.001
T4	350	15.3	27	7.0	
*N-stage*					
N0	1519	66.5	272	70.5	0.128
N1/2	764	33.5	114	29.5	
*Harvested lymph nodes*					
≥ 12 LN	2105	92.2	343	88.9	0.028
< 12 LN	178	7.8	43	11.1	
*Hospital classification*					
Colorectal cancer center	1808	79.2	280	72.5	< 0.001
Other hospitals	475	20.8	106	27.5	
*Resection group*					
Right-sided resection	1222	53.5	85	22.0	< 0.001
Left-sided resection	778	34.1	280	72.5	
Extended resection	213	9.3	19	4.9	
Transversum resection	70	3.1	2	0.5	
*Postoperative therapy*					
No adjuvant therapy according to guidelines	1292	56.6	248	64.2	0.002
Adjuvant therapy	565	24.7	95	24.6	
No adjuvant therapy in contradiction to guidelines	392	17.2	41	10.6	
No adjuvant therapy due to perioperative death	34	1.5	2	0.5	

**Fig. 1:**
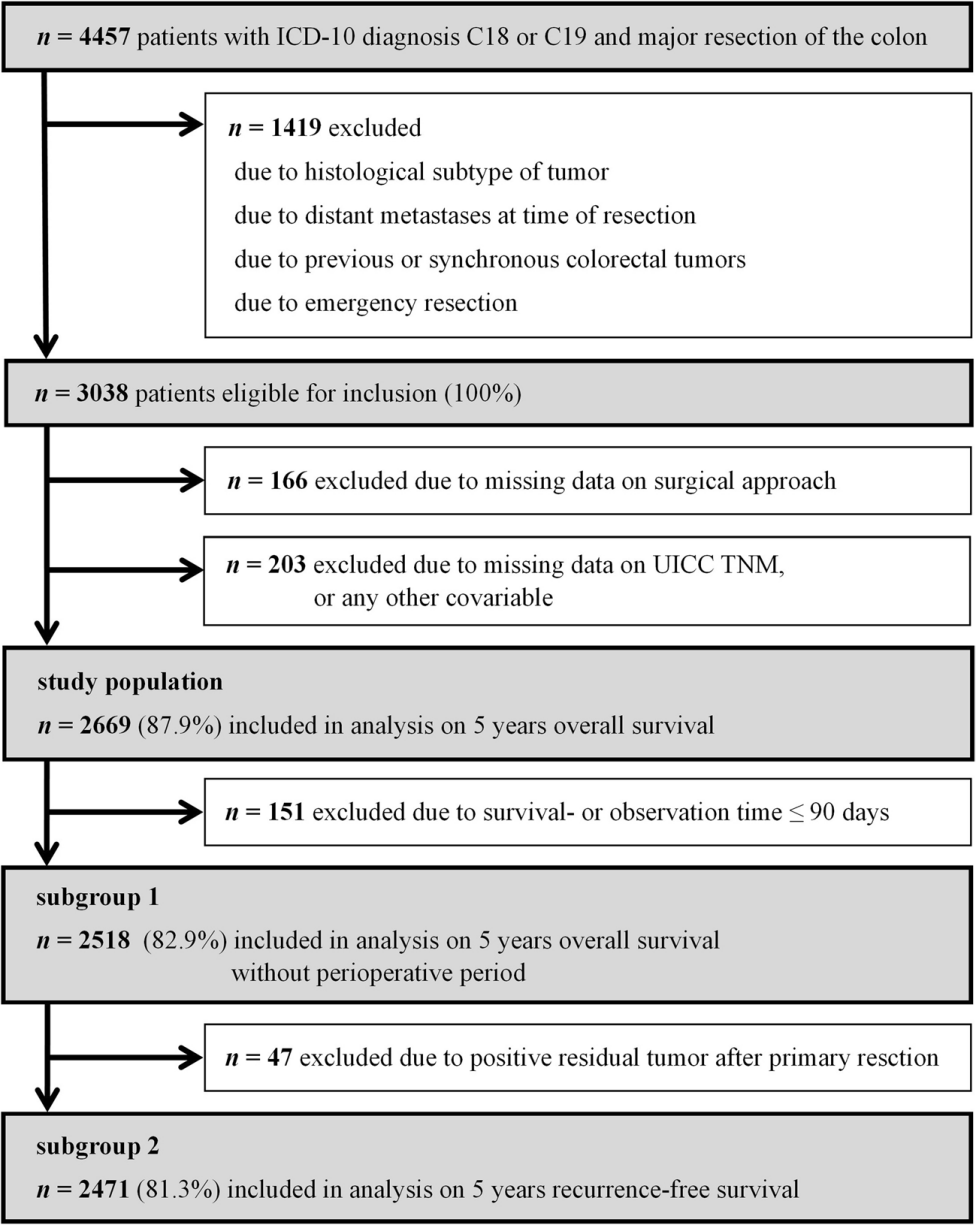
Flowchart of study patient selection

All patients with major elective resections (German Procedure Classification, OPS, 5-455, 5-456, 5-457, and 5-458 [7]) of histologically confirmed primary, non-metastatic colonic adenocarcinomas between January 1, 2004 and December 31, 2013 fulfilled the initial study inclusion criteria. Statistical requirements also render it necessary to exclude any patients with missing data. All analyses were conducted on an intention-to-treat basis, meaning that conversions remain part of the laparoscopic group. Given the median follow-up time of 6.2 years, the observation time for all analyses was restricted to 5 years. After an initial survival analysis for the entire postoperative period, a 90-day cut-off time was applied to eliminate the effect of perioperative mortality. To focus on long-term oncologic outcome, *t* = 91 days after surgery was set as the new starting point for the observation period, and patients with a survival or observation time of less than 90 days were excluded from further analyses (subgroup 1, Fig. 1). For all analyses dealing with recurrence-free survival, patients with initially positive resection margins were also excluded (subgroup 2, Fig. 1).

Apart from the usual Kaplan–Meier analyses, multivariate Cox regression models [8] were applied to account for unbalanced distribution of potential confounders. All variables with a probability of less than *p* = 0.5 in *χ* square tests of being equally distributed in the open and laparoscopic surgery groups are regarded as potential confounders which must be adjusted for. Consequently, the following variables were included in the multivariate models: gender, age, previous carcinomas (diagnosed 5 years to 3 months before the rectal tumor), synchronous carcinomas (diagnosed 3 months before to 3 months after the rectal tumor), grading, T-stage, N-stage, harvested lymph nodes, hospital classification (certified colorectal cancer center or other hospital [9, 10]), resection group, and postoperative therapy. Unlike other surveys, postoperative chemotherapy was not merely classified as “yes” or “no.” According to the current German guidelines on colorectal cancer treatment, a Union for International Cancer Control (UICC) stage I patient does not require adjuvant treatment, whereas omitting postoperative chemotherapy in stage III patients will most probably worsen their outcomes [4]. By considering the guideline recommendations when designing the variables, it was possible to form homogenous groups regarding the expected impact on patients’ health. Variable values such as “no therapy according to guidelines” or “no therapy in contradiction to guidelines” can be adjusted for in a multivariate model without the need to stratify by indication group. The R classification was never considered as part of any multivariate model, since it can be regarded as a surrogate parameter for the quality of a surgical procedure [11] rather than a confounding variable. Moreover, having adjusted for T- and N-stage separately, the inclusion of UICC stage grouping would not have added value to the model. Computing a relative survival model permits international comparison of the results and enables adjustment for temporal changes in life expectancy, age, and gender distribution. The underlying data on general mortality of the German population originate from the Human Mortality Database of the Max Planck Institutes [12]. To quantify potential bias due to exclusion of patients with missing data, a sensitivity analysis was also performed. All significance tests were two-sided with a significance level of 0.05. Results are displayed as *p* values or 95% confidence intervals (CI). The findings of this survey are presented in strict compliance with the Strengthening the Reporting of Observational studies in Epidemiology (STROBE) statement [13]. During this study, IBM SPSS 23 (IBM Corp., SPSS for Windows, Armonk, NY, USA), as well as R version 3.3.2 (R Foundation for Statistical Computing, Vienna, Austria; http://www.R-project.org/) and the R package “relsurv” (Maja Pohar-Perme [14]) were used.

## Results

Between January 1, 2004 and December 31, 2013, 4457 patients living in the observed region received surgery for colon carcinoma with radical intent. In accordance with the initially defined inclusion criteria, 1419 patients could not be considered due to distant metastases, previous or synchronous colorectal neoplasms, an unusual histologic tumor subtype, or because they had undergone an emergency procedure. Of the remaining 3038 patients, 369 were excluded on the basis of missing data on important variables (Fig. 1).

**Fig. 2:**
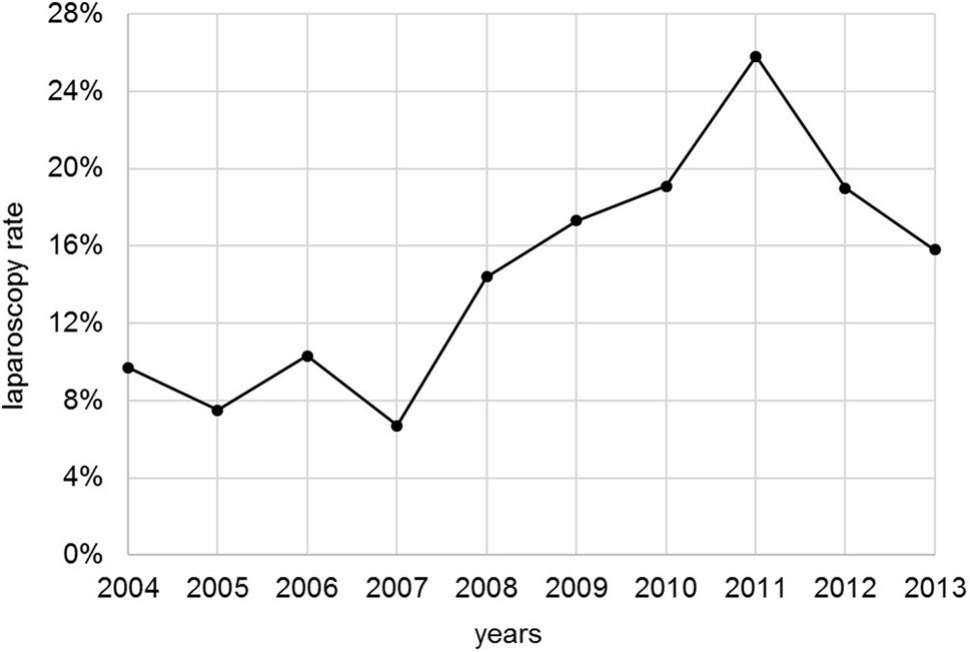
Laparoscopy rate

Among the 2669 included patients, 14.5% had received laparoscopic procedures. Between 2004 and 2011, the laparoscopy rate increased from 9.7 to 25.8%; hereafter, it dropped again to 15.8% in 2013 (Fig. 2). Of all resections, 78.2% were performed at certified colorectal cancer centers with a mean caseload of 34.8 resections per center and year. Compared to the open resection group, laparoscopy patients were younger by 4.1 years on average, with significant differences concerning the distribution of age groups (*p* < 0.001). There is also a significant difference in the proportions of UICC stages (*p* < 0.001), with a tendency toward lower T-stages (*p* < 0.001) and less lymph node metastases (*p* = 0.128) in the laparoscopic group. In 88.9% of all laparoscopic and 92.2% of all open resections, 12 or more lymph nodes were examined postoperatively (*p* = 0.028), meeting the recommendations of the German colorectal cancer treatment guideline. Of all laparoscopic procedures, 72.5% are left-sided resections, while more than every second open procedure is a right-sided hemicolectomy (*p* < 0.001). Laparoscopically treated patients were more likely to receive postoperative chemotherapy if dictated by the guidelines (*p* = 0.002; Table 1).

**Fig. 3:**
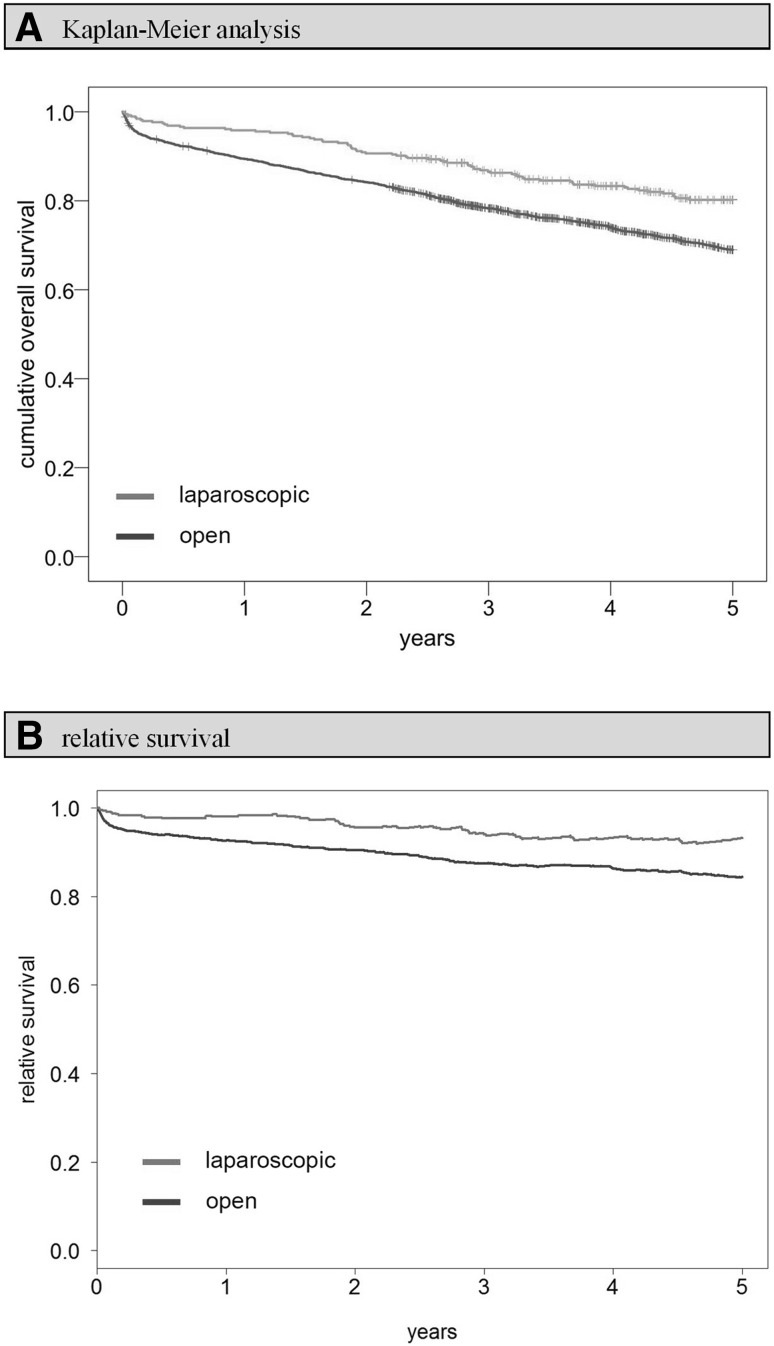
Overall survival including perioperative period (0 days–5 years). **A** Kaplan–Meier analysis: 5-year cumulative overall survival rate open versus laparoscopic: 69.0 versus 80.2%, *p* < 0.001. **B** Relative survival analysis: 5-year relative survival rate open versus laparoscopic: 84.4 versus 93.2%, *p* = 0.001

**Fig. 4:**
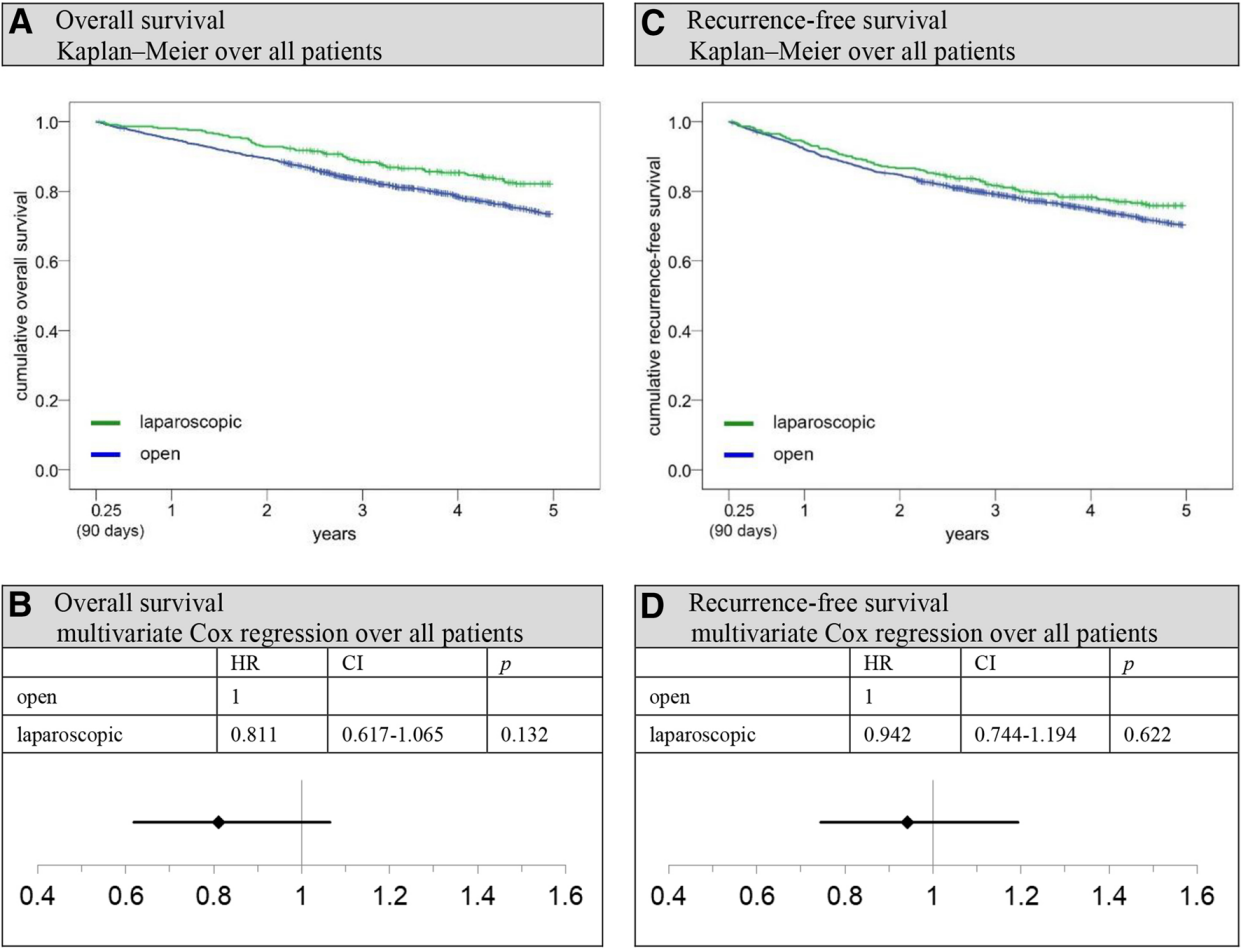
Overall and recurrence-free survival after perioperative period (91 days–5 years). *HR* hazard ratio, *CI* two-sided 95% confidence interval. **A** Kaplan–Meier analysis over all patients: 5-year overall survival rate open versus laparoscopic: 73.4 versus 82.1%, *p* = 0.001. **B** 5-year overall survival: Multivariate Cox regression analysis over all patients, adjustment for gender, age, previous carcinomas, synchronous carcinomas, grading, T-stage, N-stage, harvested lymph nodes, hospital classification, resection group, and postoperative therapy; reference: open approach. **C** Kaplan–Meier analysis over all patients: 5-year recurrence-free survival rate open vs. laparoscopic: 70.3 versus 75.9%, *p* = 0.061. **D** 5-year recurrence-free survival: Multivariate Cox regression analysis over all stages, adjustment for gender, age, previous carcinomas, synchronous carcinomas, grading, T-stage, N-stage, harvested lymph nodes, hospital classification, resection group, and postoperative therapy; reference: open approach

**Fig. 5:**
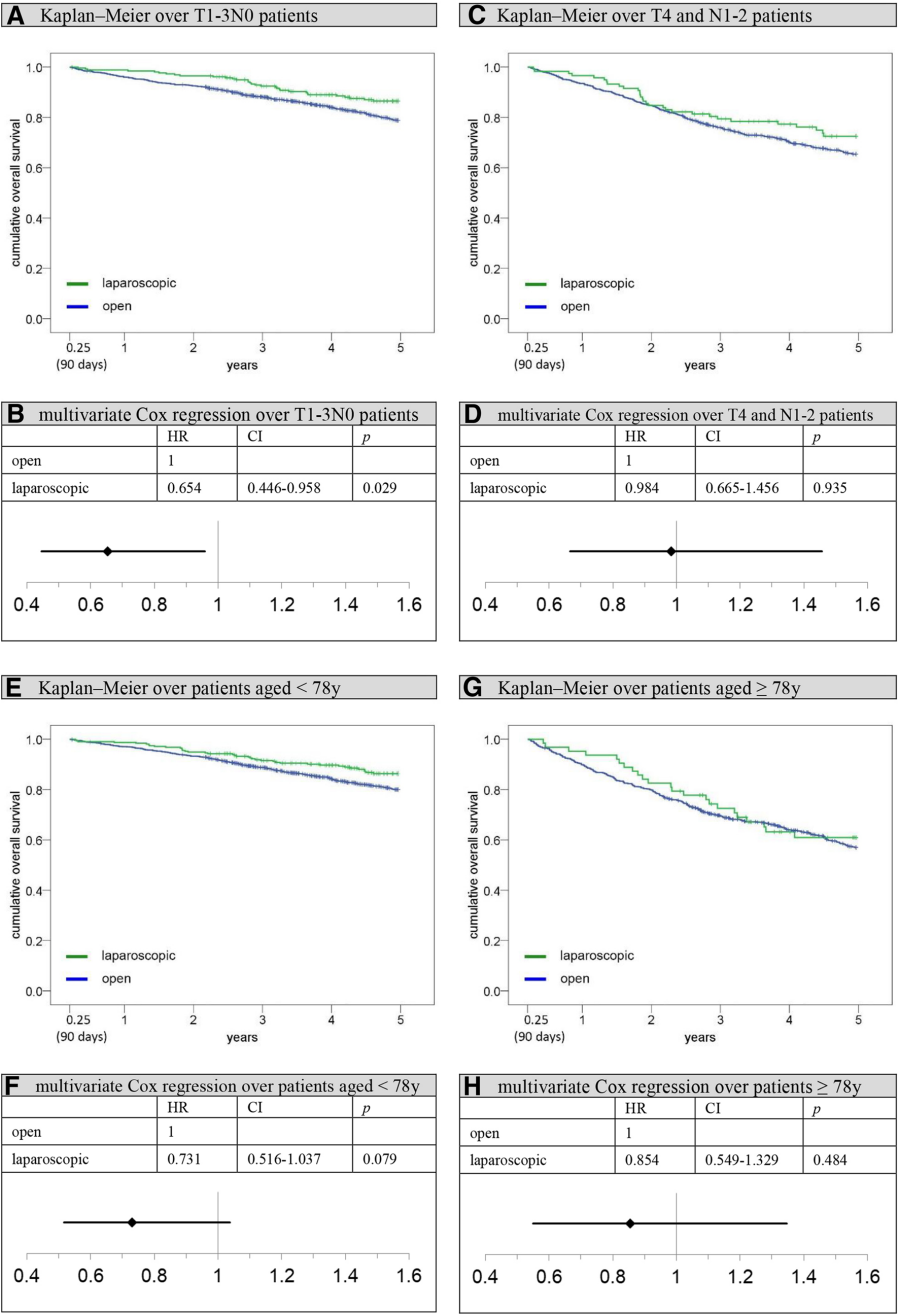
Overall survival after perioperative period (91 days–5 years), stratified analysis. *HR* hazard ratio, *CI* two-sided 95% confidence interval, *y* years. **A** Kaplan–Meier analysis over T1-3N0 patients: 5-year cumulative overall survival rate open versus laparoscopic: 78.8 versus 86.5%, *p* = 0.009. **B** Multivariate Cox regression analysis over T1-3N0 patients, adjustment for gender, age, previous carcinomas, synchronous carcinomas, grading, harvested lymph nodes, hospital classification, resection group, and postoperative therapy; reference: open approach. **C** Kaplan–Meier analysis over T4 and N1-2 patients: 5-year cumulative overall survival rate open vs. laparoscopic: 65.3 versus 72.5%, *p* = 0.167. **D** Multivariate Cox regression analysis over T4 and N1-2 patients, adjustment for gender, age, previous carcinomas, synchronous carcinomas, grading, harvested lymph nodes, hospital classification, resection group, and postoperative therapy; reference: open approach. **E** Kaplan–Meier analysis over patients aged < 78 years: 5-year cumulative overall survival rate open versus laparoscopic: 80.0 versus 86.3%, *p* = 0.016. **F** Multivariate Cox regression analysis over patients aged < 78 years, adjustment for gender, previous carcinomas, synchronous carcinomas, grading, T-stage, N-stage, harvested lymph nodes, hospital classification, resection group, and postoperative therapy; reference: open approach. **G** Kaplan–Meier analysis patients aged ≥ 78 years: 5-year cumulative overall survival rate open versus laparoscopic: 57.0 versus 60.9%, *p* = 0.569. **H** Multivariate Cox regression analysis over patients aged ≥ 78 years, adjustment for gender, previous carcinomas, synchronous carcinomas, grading, T-stage, N-stage, harvested lymph nodes, hospital classification, resection group, and postoperative therapy; reference: open approach

Comparing the Kaplan–Meier overall survival rates of open and laparoscopically treated patients 5 years after surgery, there was a benefit for the latter group (open vs. laparoscopic: 69.0 vs. 80.2%; *p* < 0.001; Fig. 3). The situation did not change much when comparing 5-year relative survival rates (open vs. laparoscopic: 84.4 vs. 93.2%; *p* = 0.001; Fig. 3). Setting *t* = 91 days after surgery as the new starting point and excluding all patients who died perioperatively or whose observation time was shorter than 91 days (subgroup 1, Fig. 1), the 5-year overall survival rate was 73.4% for open surgery and 82.1% for laparoscopy patients (*p* = 0.001). Significant advantages for laparoscopy could be seen particularly in stages T1-3N0 (5-year overall survival rate open vs. laparoscopic: 78.8 vs. 86.5%; *p* = 0.009; Fig. 5) and in patients aged younger than 78 years (5-year overall survival rate open vs. laparoscopic 80.0 vs. 86.3%; *p* = 0.016; Fig. 5). A multivariate Cox regression analysis was conducted to objectify these results. Applying the previously described methodology to control for all potentially unequally distributed confounders, it was adjusted for the factors stated above. Thereafter, a survival benefit for laparoscopically treated patients was still observed, although the significance level was no longer reached (hazard ratio (HR) 0.811; 95% CI 0.617–1.065; *p* = 0.132; Fig. 4). However, the stratified analysis showed that laparoscopy produces significantly superior adjusted outcomes in stage T1-3N0 patients (HR 0.654, 95% CI 0.446–0.958; *p* = 0.029; Fig. 5). No significant differences between the two surgical approaches could be observed in T4 tumors or patients with lymph node metastases. Examining elderly (≥ 78 years) and younger patients separately, no significant differences were seen between laparoscopy and laparotomy either. However, with patients aged 77 years and younger, the survival benefit for laparoscopic patients only narrowly missed the significance level. Sensitivity analysis indicated that the necessary exclusion of patients with missing data did not favor the laparoscopic group, since excluded open surgery and laparoscopy patients showed comparable survival rates (5-year overall survival rate open-excluded vs. laparoscopic-excluded: 53.8 vs. 55.3%; *p* = 0.646).

Evaluating recurrence-free survival rates generated similar but not identical results. The following analyses were all restricted to an observation time starting at *t* = 91 days and patients with no residual tumors (subgroup 2, Fig. 1). The 5-year recurrence-free Kaplan–Meier survival rate is 70.3% in the open and 75.9% in the laparoscopic group, with the significance level narrowly missed (*p* = 0.061; Fig. 4). In multivariate Cox regression analysis, laparoscopy retained its superiority; however, the actual effect size was reduced even more (HR 0.942, 95% CI 0.744–1.194; *p* = 0.622; Fig. 4). Sensitivity analysis showed that excluded open surgery and laparoscopy patients had similar recurrence-free survival rates (5-year recurrence-free survival rate open-excluded vs. laparoscopic-excluded: 52.4 vs. 53.3%; *p* = 0.988). Therefore, the superior laparoscopy group was not favored by the exclusion process.

## Discussion

Laparoscopic procedures have been performed for oncologic diseases of the lower intestinal tract for many years. According to a systematic Cochrane review published in 2012 by Kuhry et al., “laparoscopic resection of carcinoma of the colon is associated with a long-term outcome no different from that of open colectomy” [3]. Recently, Deijen et al. reported the 10-year follow-up of the COLOR I trial and also came to the conclusion that “Laparoscopic surgery for non-metastatic colon cancer is associated with similar rates of disease-free survival, overall survival and recurrences as open surgery” [15]. Different representative trials from the Netherlands or the UK report laparoscopy rates exceeding 50%, whereas in other countries like Sweden or Germany, the proportion of laparoscopic procedures is often less than 20% [16–19]. As far as the region observed in this study is concerned, medical underdevelopment does not seem to be the reason for low or even decreasing laparoscopy rates: three out of four resections were performed at specialized colorectal cancer centers. Obviously, doubt exists concerning whether the non-inferiority of laparoscopy proclaimed by many RCTs conducted in ambitious hospitals under ideal circumstances can be transferred to daily clinical practice. To answer this question, large observational studies are required. Unfortunately, truly population-based surveys on the topic are scarce. Although some large studies of this type are available from the United States, Canada, or the Netherlands, these focus exclusively on short-term outcomes. For example, Zheng et al. used the data of approximately 50,000 patients from the US National Cancer Database and reported a lower readmission rate after laparoscopic resections (laparoscopic 4.8% vs. open 5.5%; *p* = 0.003). These authors also observed a significantly lower perioperative mortality rate for laparoscopically treated patients (odds ratio (OR) 0.59; 95% CI 0.49–0.69 [20]). McKay et al. and Kolfschoten et al. used smaller but nevertheless representative study populations, and observed similar results [21, 22]. Apart from these studies, only a few trials deal with long-term survival. More than 10 years ago, Kube et al. conducted pioneering work and published results from a large German observational trial, reporting 5-year overall and tumor-free survival rates. The latter authors observed a substantial advantage for laparoscopy patients, with results quite close to the outcomes presented in this study (5-year overall survival rate open vs. laparoscopic: 66.9 vs. 82.8%; *p* = 0.005 [23]). It must, however, be mentioned that all hospitals participating in this study did so voluntarily and loss to follow-up seemed to play a substantial role. In 2016, Stormark et al. published their findings on long-term survival after laparoscopic colon surgery, with favorable results for the new approach [24]. These authors had at their disposal a considerable case number with an acceptable exclusion rate, used highly representative data from the Norwegian Colorectal Cancer Registry, and applied reasonably transparent statistical methods. In the same year, Benz et al. took data from 30 German cancer registries and compared long-term survival of open and laparoscopic colon cancer surgery up to a 10-year observation time [19]. Benz et al. also regarded laparoscopy to be a safe alternative to the open approach, although they excluded, for various reasons, a considerable number of patients who would generally have been eligible for inclusion; the external validity of these findings thus remains unclear to some degree.

Acknowledging the achievements and most common problems of previous publications, the current study aimed to minimize the risk of any form of bias. Before excluding any patient because of missing data, all possible measures to fill information gaps were undertaken. During this process, the four-eyes principle was always applied to match the high-quality standards of the Tumor Center Regensburg. If information on an important item was not available, exclusion of the patient was ultimately unavoidable. In order to quantify a potential bias caused by the exclusion process, a sensitivity analysis was performed. Interpreting the results of the sensitivity analysis, the conclusion can be reached that the presented outcomes are stable and not biased by missing data. Retrospective surveys such as the current analysis use data straight out of daily clinical routine and thus truly depict reality. On the downside, it has to be accepted that comparison groups are not created at random, which means that an indication bias always exists. Steele et al. could, for example, show that younger age and lower tumor stages are significant predictors for the selection of a laparoscopic approach [25], a result which is confirmed by the present study. Multivariate Cox regression analysis allows for correction of different risk profiles between groups. By adjusting for variables such as hospital status, tumor stage, or additional therapies, it was possible to cover a lot of inhomogeneities. One may question though, if it was justified to include the number of harvested lymph nodes as a covariable in the multivariate model. After all, there is a strong association between surgical quality and adequate lymphadenectomy [26, 27]. Adjusting for an associated survival benefit [28, 29] therefore disadvantages the superior surgical approach. On the other hand, if pathological examination tended to be less thorough with specimens of one approach, systematic understaging and worse survival rates would be the consequence. In order to avoid the latter problem, the harvested lymph nodes were finally included in the model. Unfortunately, there was no information available concerning non-oncologic comorbidities. This is probably the most important limitation of this survey, since weaker patients are more likely to die from strenuous treatment side effects or other conditions not directly associated with their tumor [30]. After all, cardiopulmonary problems are the most common cause of death, even in front of neoplastic diseases [31]. However, evidence exists that adjustment for age partially includes adjustment for comorbidities. The older a patient is, the more potentially life-threatening illnesses he or she suffers from [32]. According to survey of the Dutch Cancer Registry on colorectal cancer patients, there is a significant association between age and the number of a person’s comorbidities [33]. Notwithstanding this, systematic documentation of ASA or a different comorbidity score is required to be able to conduct an even more accurate risk adjustment.

Regardless of the statistical methods applied, the question of whether certain patient subgroups may benefit more from laparoscopy than others remains. Within the setting of this study, overall survival of patients with less to moderately invasive carcinomas was significantly positively influenced by laparoscopy. This supports the findings of the previously mentioned COLOR I trial, where the largest survival benefit after 5 years of observation time could be seen among UICC stage II patients, although the significance level was not reached [34]. Younger patients also seem to benefit from laparoscopic surgery, although the significance level in multivariate analysis is missed only narrowly. Whereas laparoscopy in low-risk situations can be recommended without restrictions, a closer look has to be taken in more advanced tumor stages and elderly patients. There is a certain suspiciousness concerning whether laparoscopy may be used with T4 patients. In the course of the COLOR I trial, half of the T4 patients undergoing laparoscopic surgery required conversion [34], which may be associated with worse survival [35, 36]. Thereafter, different publications addressed the topic with generally favorable outcomes for laparoscopy [37, 38]. The present study could also demonstrate that, even in high-risk situations like T4 or lymph node-positive tumors, laparoscopy is a non-inferior alternative. Moreover, it can add to the evidence that minimally invasive surgery should be considered for elderly patients, too [21, 39–42]. Old people benefit from short-term advantages such as shorter hospital stays or lower complication rates like no other age group, without the need to fear a negative impact on their limited life expectancy.

## Conclusion

While in some regions laparoscopy rates for colon carcinoma resections remain static at a low level, this study reports favorable long-term outcomes after minimal invasive surgery, especially for less invasive tumors. Even in high-risk situations, laparoscopy produces results equal to open surgery. Therefore, this study confirms the external validity of previous RCTs under routine clinical conditions. The further implementation of systematic tumor documentation in countries without comprehensive cancer registries would allow for more population-based studies on the topic and thus contribute to a higher evidence level.
